# Vitamin D status in cord blood and newborns: ethnic differences

**DOI:** 10.1186/1824-7288-39-35

**Published:** 2013-06-04

**Authors:** Francesco Cadario, Silvia Savastio, Erica Pozzi, Antonella Capelli, Elena Dondi, Miriam Gatto, Mauro Zaffaroni, Gianni Bona

**Affiliations:** 1 Division of Pediatrics, Department of Health Sciences, University of Piemonte Orientale, Novara, Italy; 2 IRCAD, (Interdisciplinary Research Center of Autoimmune Diseases), Novara, Italy

**Keywords:** Vitamin D, Newborn, Ethnicity, Skin colour

## Abstract

**Background:**

A deficiency in vitamin D (25OHD) is common throughout the world in both adults and children, being related to skin pigmentation, sun exposure, dietary intake and obesity. Limited data are available for the neonatal age. The aim of the study is to understand the differences in 25OHD levels with respect to skin colour and ethnicity in newborns.

**Methods:**

We randomly enrolled 62 neonates, born at term and appropriate for gestational age. Thirty two were born from Italian mothers with fair skin (FS) and 30 from non-Caucasian mothers (North African, African, Asian and Latin American): 10 with light olive/light brown (LOB) and 20 with medium brown/black skin (MBB). Vitamin D was measured in the cord blood at birth and in neonatal serum during metabolic screening.

**Results:**

25OHD levels were (mean ± SD) 21.4 ± 11 ng/ml in cord blood and 14.9 ± 7 ng/ml in serum after birth. 25OHD values were higher in cord blood (p < 0.01) and neonatal serum (p < 0.001) in subjects supplemented with Vitamin D. Newborn FS showed higher vitamin D levels in cord blood when compared to LOB and MBB (p < 0.01), and higher levels in neonatal serum when compared to LOB (p < 0.01). In cord blood, 25OHD levels were higher in Italian newborns than in North African (p < 0.004) and African (p < 0.01). In neonatal serum, 25OHD levels were higher in Italian infants only when compared with North African infants (p < 0.03).

**Conclusions:**

The present study shows a high prevalence of vitamin D insufficiency and deficiency in newborns with significant differences observed to be due to ethnicity, skin colour and maternal supplementation during the pregnancy.

## Background

Vitamin D is a steroid hormone with multiple actions on most tissues in the body. It comes in two forms: ergocalciferol (vitamin D2) present in plants and specific types of fish and cholecalciferol (vitamin D3), synthesized in the skin by 7-dehydrocholesterol through UV radiation. Vitamin D3 is the main source of vitamin D for humans. Vitamin D that comes from the skin or diet is biologically inert and must be first hydroxylated in the liver (25OHD), with a further hydroxylation in the kidney (1,25OH2D) to be biologically active. Circulating 25OHD is the major circulating form of vitamin D and is considered as the primary indicator of vitamin D status [1].

1,25OH2D stimulates intestinal calcium absorption and regulates this function in a wide number of other tissues. Moreover, vitamin D is a modulator of the innate and adaptive immune system and prospective observational studies suggest that vitamin D supplements in infancy and early childhood may decrease the incidence of chronic diseases such as type 1 diabetes mellitus [1,2]. Current data within the literature demonstrates a high prevalence of vitamin D insufficiency not only in adult and in children, but also in pregnant women and in their neonates [3-5]. Risk factors for vitamin D deficiency and rickets in early life include breast-feeding without vitamin D supplementation, dark skin pigmentation, season, latitude and maternal vitamin D deficiency [1]. The latest guidelines define as insufficient serum levels below 30 ng/mL (72.5 nmol/liter) and as deficient, levels below 20 ng/mL (50 nmol/liter) in both adults and children [1-6]. To date, specific range for neonates is not available.

In utero, the fetus is wholly dependent on the mother for vitamin D. The 25OHD crosses the placenta into the blood stream of the fetus with a half-life of approximately 2 months [7]. However, most pregnant women are vitamin D deficient or insufficient with a consequential deficiency or insufficiency in their neonates [5-8]. Bodnar et al. [9], in a study of 200 white and 200 black mother-infant pairs, reported that more than 50% of mothers and newborns had a vitamin D insufficiency, despite the use of multivitamins in pregnancy. Population studies in Europe have shown that immigrants from Asia, the Middle East or Africa have a greater risk of low vitamin D levels [10-12]. Moreover, some studies indicate that ethnic differences play a role in the circulating levels of serum vitamin D [13-15]. Dark-skinned individuals require a greater duration of exposure (4–5 times) than their light-skinned counterparts to synthesise comparable amounts of vitamin D [16]. Country of origin, genetic traits and cultural behaviour are important factors in determining vitamin D levels [17].

Currently, the literature shows that neonatal vitamin D status at birth is highly correlated with maternal vitamin storage. Data suggests that doses exceeding 1000 IU of vitamin D per day are necessary to achieve sufficienct 25OHD concentrations in pregnant women [18]. Discordant data are present with respect to the role of vitamin D supplementation during pregnancy. Supplementation with D3 instead of D2 has greater efficacy in raising circulating 25OHD concentrations, but only few studies show statistically significant differences in cord blood 25OHD levels between infants of mothers supplemented versus non-supplemented. Further, multivitamin use during pregnancy has been demonstrated to not be sufficient to correct pre-existing maternal vitamin D deficiency [19-22].

In order to understand the differences in vitamin D levels depending on skin colour and ethnic differences, we evaluated 25OHD levels at birth in cord blood and in the first three days of life from ethnically diverse infants born within our Hospital.

## Materials and methods

### Subjects

Sixty-two infants born at term (37–41 weeks of gestation), appropriate for gestational age (AGA), were enrolled. AGA was defined as a birth weight from 10th to 90th percentile for gestational age according to Italian charts [23]. All babies were born from vaginal delivery, after uncomplicated pregnancies and were otherwise healthy. None of the babies showed signs of distress at delivery.

Thirty-two infants were born to Italian mothers with very fair/fair skin and 30 to non-Caucasian mothers with dark skin: 10 with light olive/light brown and 20 with medium brown/black skin. Ten mothers were North African, 10 Central and Southern African, 5 Asian and 5 Latin American. North African mothers had light olive/light brown skin, all others medium brown/black skin.

All newborns and their mothers were randomly enrolled within Division of Pediatrics, University of Piemonte Orientale, Novara, Italy from June to September 2009. The study protocol was approved by the responsible Ethics Committee of Novara and informed consent was obtained by newborn’s parents before the sample collections.

All the mothers were healthy, in particular none had chronic diseases. Exclusion criteria were the presence of any organic diseases in particular neurological, endocrine, liver and kidney abnormalities in the mother or in the newborns.

Birth weight and length were recorded at birth by the attending nurse. All data was collected appropriately.

Newborns showed (mean ± SD) a birth weight of 3252 ± 426 g, a length of 54.7 ± 3 cm and a gestational age of 39.1 ± 1.2 weeks. All infants had an Apgar score of 8–10 points at 1 and 5 minutes. None had neonatal distress or intrapartum complications.

### Maternal supplementation

The type and duration of maternal supplementation with Vitamin D was investigated during hospitalization. All mothers were considered adequately supplemented, with an intake of 400 IU vitamin D per day as recommended by The Institute of Medicine.

All mothers had used daily a standard prenatal multivitamin containing 400 IU of vitamin D (vitamin D3), except for 4 mothers (2 times per day). Pure vitamin D preparations were not used in any case.

Twenty of 32 Italian mothers and 15 of 30 non Caucasian mother were supplemented during pregnancy. The average daily vitamin D intake was 420 IU ± 80 IU/d and the average duration of supplementation was 117 ± 30 days. All mothers assumed regularly the supplementation also in the last third trimester of pregnancy.

### Hormonal parameters

25OHD was investigated in cord blood at delivery and in neonatal serum by venous blood sampling. Sampling was performed in the first three days of life during the neonatal metabolic screening.

25OHD levels (ng/ml) were measured by LIAISON^®^, (DiaSorin, Inc, Stillwater, MN). The LIAISON 25 OH Vitamin D TOTAL Assay uses chemiluminescent immunoassay (CLIA) technology for the quantitative determination of 25OHD and other hydroxylated vitamin D metabolites in human serum. The analytical measurement range for the DiaSorin LIAISON 25 OH Vitamin D Total Assay is 4 ng/mL to 150 ng/mL and the sensitivity is 4 ng/mL.

Data are expressed as mean ± SD. For continuous variables, the variation between groups was compared by means on nonparametric Wilcoxon test. A correlation analysis was performed using the Pearson’s correlation test with logarithmic transformations where necessary. A stepwise regression model with two-tailed probability values was used to measure the strength of association between variables. Statistical significance was assumed for p < 0.05. All statistical analyses were performed with SPSS for Windows version 17.0 (SPSS INC; Chicago, IL, USA).

## Results

The mean vitamin D levels in all subjects were 21.4 ± 11 ng/ml on cord blood and 14.9 ± 7 ng/ml in neonatal serum.

**Figure 1:**
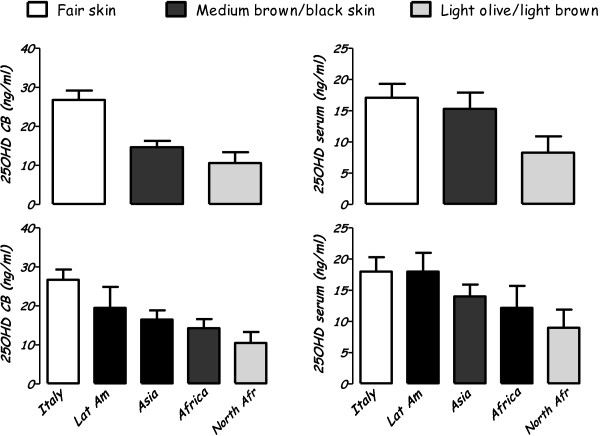
Vitamin D levels (21OHD) in cord blood and serum levels by skin colour and ethnic origin.

25OHD values were analysed by skin colour and by the mothers’ country of origin (Figure 1). No sex differences were found.

In cord blood, 25OHD values were <20 ng/ml in 30 cases (55.5%): 18 (33.3%) had mild vitamin D deficiency (between 10 and 20 ng/ml) and 12 (22.2%) had severe deficiency (<10 ng/ml).

In newborns, vitamin D levels were deficient (<20 ng/dl) in 75.6% of cases, with a severe deficiency (<10 ng/ml) in 46.3% and a mild deficiency (10–20 ng/ml) in 29.3%.

Analyzing exclusively infants born to immigrant mothers, none demonstrated 25OHD >30 ng/ml in either cord blood or in neonatal serum. A deficiency in Vitamin D levels was found in the cord blood of 77.3% cases and in 81,8% of neonatal serum with a severe deficiency in 36,4% and 54,5%, respectively.

When considering the maternal multivitamin intake during pregnancy, all mothers supplemented with vitamin D demonstrated higher 25OHD values in both cord blood (24.5 ± 11.9 vs 17.2 ± 9.2 ng/ml; p < 0.01) and in neonatal serum (17.7 ± 9.3 vs 11.1 ± 6 ng/ml; p < 0.001).

### 25OHD levels analysed by skin colour

Vitamin D levels on cord blood were higher in neonates with fair skin (FS) than with dark skin (p < 0.0001), both with light olive/light brown (LOB) (p < 0.01) or medium brown/black skin (MBB) (p < 0.01).

Three days after birth, newborns FS showed higher 25OHD levels only when compared with LOB (p < 0.01). MBB had serum 25OHD values higher than LOB (p < 0.02) without significant differences in cord blood (Table 1).

**Table 1:** Vitamin D levels on cord blood (25OHD CB) and neonatal serum (25OHD N) by skin colour

	***FS***	***Dark skin***	
		***LOB***	***MBB***
	**32**	**10**	**20**
***GA***	38.8 ± 1.1	39.5 ± 1.1	39.5 ± 1.2
***BW***	3175 ± 455	3420 ± 539	3303 ± 318
***25OHD CB***	26.8 ± 13 ^a,b^	10.6 ± 6.9^a^	14.7 ± 6.2^b^
***25OHD N***	17.1 ± 8^a^	8.3 ± 4^a,c^	15.3 ± 8^c^

Abbreviations: *GA *gestational age, *BW *Birth weight, *FS* very fair/fair skin, *LOB *light olive/light brown, *MBB *medium brown/black skin. a: p < 0.01 FS vs LOB; b: p < 0.01 FS vs MBB; c: p < 0.02 MMB vs LOB.

Differences according to maternal supplementation, were shown only in the MBB group (p < 0.01) with higher vitamin D levels in cord blood and neonatal serum with supplementation. No differences with the FS and LOB groups were found.

In cord blood, 25OHD levels were higher in FS than LOB (p < 0,004) and MBB (p < 0.04) regardless of maternal supplementation.

Neonates FS with no supplementation during pregnancy, showed vitamin D levels higher than unsupplemented LOB and MBB neonates (p < 0.05). No differences were found in case of maternal supplementation in pregnancy (Table 2).

**Table 2:** Vitamin D levels on cord blood (25OHD CB) and neonatal serum (25OHD N) according to maternal supplementation (S) in pregnancy

	***FS***		***LOB***		***MMB***	
	**S 20**	**No S 12**	**S 5**	**No S 5**	**S 10**	**No S 10**
***25OHD CB***	29.2 ± 11.4^a,b^	22.8 ± 12^a,b^	11.9 ± 6.8^a^	4.2 ± 0.5^a^	19.4 ± 6.4^b^	11.9 ± 5.4^b^
***25OHD N***	18.0 ± 7.9	15.9 ± 9^c^	11.3 ± 4.0	4.2 ± 0.3^c^	20.1 ± 11	8.2 ± 2.3^c^

Abbreviations: *FS *very fair/fair skin, *LOB *light olive/light brown, *MBB *medium brown/black skin, *S *mothers supplemented with Vitamin D3, *Non S *mothers not supplemented with Vitamin D3. a: p < 0.004 FS vs LOB; b: p < 0.04 FS vs MMB; c: p < 0.04 FS vs LOB and MMB.

### 25OHD levels analysed by mothers’ country of origin

The cord blood from italian newborns (ItaN), showed 25OHD levels higher than North African (NAN) (p < 0.004) and African (AN) (p < 0.01).

At three days of life, vitamin D values were higher in ItaN only when compared with NAN (p < 0.03). NAN had 25OHD levels lower than AN (p < 0.02) without significant differences in cord blood levels.

No differences in Vitamin D levels were shown between ItaN and neonates born from Asian or Latin American mothers (Table 3).

**Table 3:** Vitamin D levels on cord blood (25OHD CB) and neonatal serum (25OHD N) by mothers’ country of origin

	***Italy***	***North Africa***	***Africa***	***Asia***	***Latin America***
	**32**	**10**	**10**	**5**	**5**
***GA***	38.8 ± 1.1	39.5 ± 1.1	39.7 ± 0.9	38.8 ± 1.6	40 ± 1.1
***BW***	3175 ± 455	3420 ± 539	3425 ± 300	3039 ± 244	3291 ± 279
***25OHD CB***	26.8 ± 13^a,b^	10.6 ± 6.9^a^	14.3 ± 7^b^	16.5 ± 4.9	19.5 ± 10
***25OHD N***	17.1 ± 8^c^	8.3 ± 4^c,d^	14 ± 5.7^d^	12.2 ± 7	19.3 ± 10

Abbreviations: *GA *gestational age, *BW *Birth weight; a: p < 0.004 ITALY vs NORTH AFRICA; b: p < 0.01 ITALY vs AFRICA; c: p < 0.03 ITALY vs NORTH AFRICA; d: p < 0.02 AFRICA vs NORTH AFRICA.

### Correlations

Vitamin D levels correlated in cord blood samples and neonatal serum (r: 0.836, p < 0.0001), even when corrected for months, gestational age and birth weight.

Gestational age not correlated with cord blood vitamin D or neonatal serum levels.

Multiple regression analysis revealed that vitamin D levels in cord blood were predicted (R2: 0.670; p < 0.0001) by neonatal Vitamin D values (standardized β: 0.818), when corrected for vitamin D supplementation and gestational age. Likewise neonatal vitamin D levels were predicted (R2: 0.670; p < 0.0001) by Vitamin D values on cord blood (standardized β: 0.818), when corrected for vitamin D supplementation and gestational age.

## Discussion

The present study demonstrates a high prevalence of vitamin D deficiency and insufficiency both in cord blood and in neonatal serum, with lower concentrations in infants born from immigrant mothers.

Previous studies have shown a high prevalence of vitamin D deficiency among neonates and their mothers, particularly in United States, Australia, South Asia, the United Kingdom, Greece and in other European countries [24-29]. However, there is no absolute consensus on which is the normal range for 25OHD in newborns.

Analysing data by skin colour, we found that in cord blood, FS showed 25OHD levels higher than LOB or MBB neonates, while in neonatal serum FS had values higher only with respect to LOB, with no significant differences with MBB. LOB had lower serum levels with respect to MBB, suggesting a particular role for different skin colour in metabolism and in vitamin D levels.

The two major sources of vitamin D in the body are diet and the skin. The different skin colour influences the vitamin D levels with the currently available data suggesting that plasma vitamin D was lower in black neonates than white neonates [8-11].

Analyzing data by mothers according to their country of origin, 25OHD cord blood levels were higher in neonates born from Italian mothers than from North African or African mothers, while after birth vitamin D serum levels were higher in Italian newborns only when compared with North African.

Other studies have shown results, similar to ours in that, 25OHD levels reflect racial origin. Sulaiman et al. [30], measured 25OHD levels in umbilical cord blood taken from 54 white and 22 south Asian neonates in the UK during summer, showing lower serum levels in South Asian (19.8 ± 22) infants, with respect to caucasians (43.3 ± 23; p < 0.0002). Recently, Dror et al. [31], showed mean serum vitamin D levels were significantly lower in African-American than in non –African American women and their infants.

To achieve similar levels of 25OHD, subjects with dark skin and consequent high-melanin content must be exposed to UVB light 4–5 times as long as individuals with white skin and low melanin content. Recently Sloka et al. [32] in Newfoundland, a Canadian island, showed a reduction of the solar radiation and a gradient of average daily UVB of 10,5% for a change of latitude from 46°N to 52°N. Vitamin D insufficiency is common for latitudes north of 42°N, which includes Novara.

Moreover, we observed higher vitamin D levels in cord blood than in neonate serum with a strong correlation to each other. During the first 2 months of life, neonatal and maternal 25OHD levels are correlated [16,33,34].

We have also investigated the role of supplementation in pregnancy on vitamin D cord blood and serum levels. Analyzing all subjects together, mothers supplemented with vitamin D showed 25OHD values higher than mothers not supplemented both on cord blood and neonatal serum. Analyzing the groups separately for skin colour, the supplementation seemed to determine significant differences only into MMB group with higher levels on cord blood and neonatal 25OHD serum in case of supplementation. No differences were found within FS and LOB group suggesting a probably different function of the supplementation according to skin colour.

Cord blood vitamin D levels were higher in FS than LOB or MBB regardless of supplementation. After birth only FS newborns unsupplemented showed vitamin D levels higher than LOB and MBB; no differences were found in case of maternal supplementation in pregnancy, most likely due to a specific role of supplementation in neonates.

The numbers were too small for a separate analysis into each ethnic groups.

Although several studies are available on vitamin D supplementation during pregnancy, its appropriate dose is not clear [19-21]. IOM recommends during pregnancy a dietary allowance of 600 IU/day and an estimated average requirement of 400 IU/day [6]. A recent Cochrane on vitamin D supplementation during pregnancy showed that with supplementation, there is an increase serum 25OHD levels in neonates [22].

Our study has several limitations. We lack of information on several potential confounders, in particular individual sun exposure and standardization of phototype. The skin colour has been evaluated, even if by the same person, only subjectively and not confirmed by Fitzpatrick Skin type chart, one of the objective methods of standardization. Moreover, vitamin D is influenced by seasonability and the study was conducted in a single season; however, the data was collected in summer when better vitamin D levels are demonstrated by literature [1]. Another important limitation is to have enrolled few Caucasian and non Caucasian subjects to perform a valid subanalysis according supplementation and country of origin. The current data is preliminary and is the basis of an ongoing study.

Further studies are necessary to understand what component is more important between skin colour and ethnicity.

## Conclusions

The present study demonstrates a high prevalence of vitamin D deficiency and insufficiency both in cord blood and in neonatal serum with significant differences due to ethnicity, skin colour and presence or absence of supplementation. Identifying vitamin D deficiency at birth is essential for the development of public policy for prevention and supplementation.

## Abbreviations

25OHD: Vitamin D; FS: Fair skin; LOB: Light olive/light brown skin; MBB: Medium brown/black skin; ItaN: Italian Neonates; NAN: Neonates born by North African mothers; AN: Neonates born by African mothers.

## Competing interests

The authors declare no conflict of interest. There is not conflict of interest that could be perceived as prejudicing the impartiality of the research reported.

## Authors’ contributions

Francesco Cadario participated in the study design and chose eligible patients. Savastio Silvia participated in the design of the study and carried out the samples analysis. Erica Pozzi participated in the design of the study and managed the literature search. Antonella Capelli participated in the design of the study and collected the samples. Elena Dondi participated in the design of the study and managed the literature searches. Miriam Gatto participated in the design of the study and carried out the samples analysis. Mauro Zaffaroni participated in the design of the study and helped to draft the manuscript. Gianni Bona conceived the study and participated in its coordination and helped to draft the manuscript. All authors read and approved the final manuscript.
